# Supplementary material for: Bayesian MEG time courses with fMRI priors
Source: Brain Imaging Behav. 2021 Sep 25;16(2):781–91. doi: 10.1007/s11682-021-00550-4 (PMC9007727; doi:10.1007/s11682-021-00550-4)
Supplement: Supplementary file 1 — Supplementary file1 (DOCX 24092 KB) [file 11682_2021_550_MOESM1_ESM.docx]

^[[1]](#footnote-1)^ **Supplementary materials**

Fig.S1


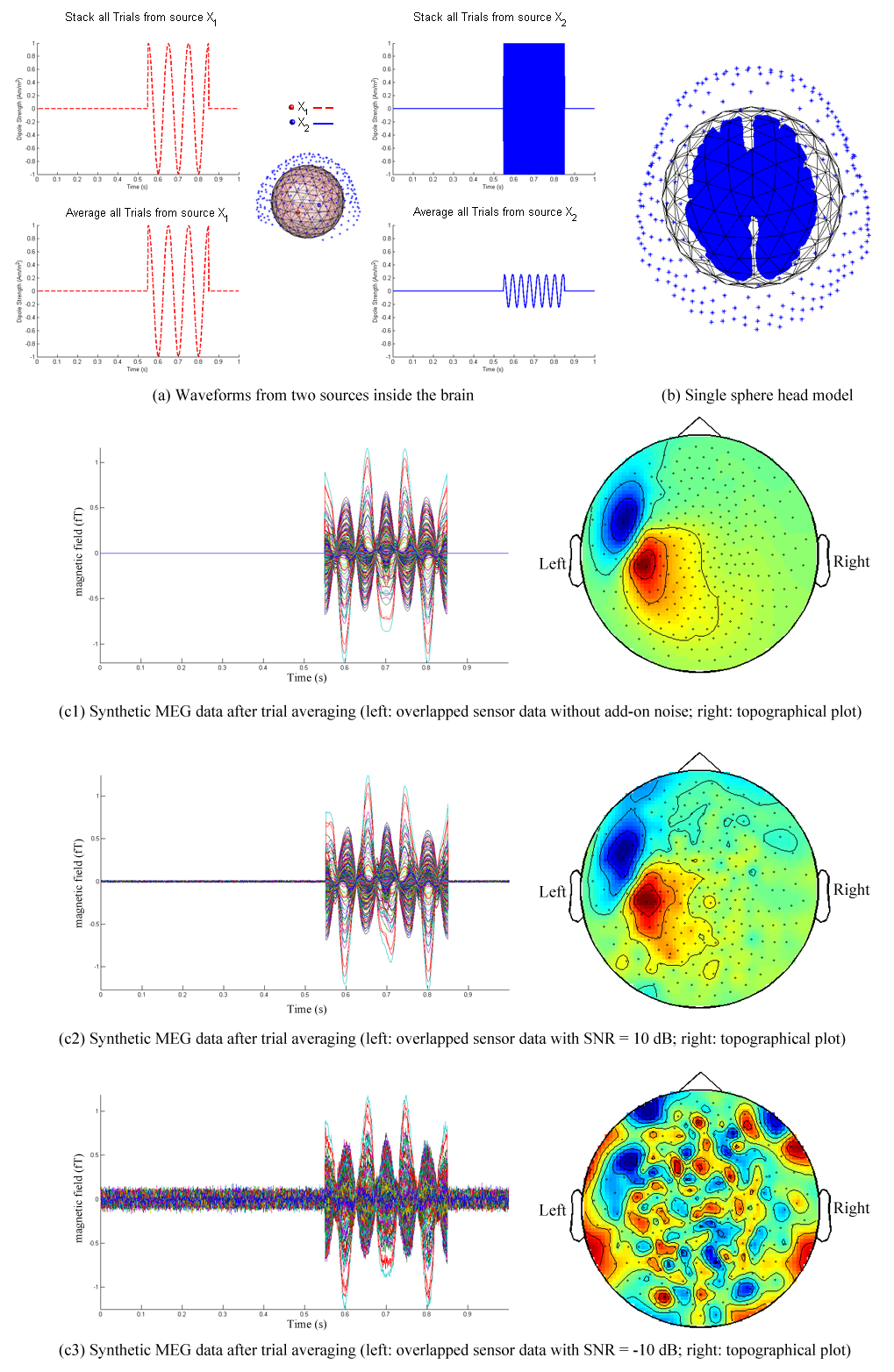
Illustration diagram showing MEG data simulation process

Fig. S2. Timing diagram of the event-related story-processing task for MEG and fMRI task. MEG: 15 5-s Trials for each condition are recorded. FMRI: 15 36-s cycles for each phase of the paradigm are presented for a total scan time of 9 minutes. Online performance data of correctness and response time are recorded for both MEG and fMRI sessions.


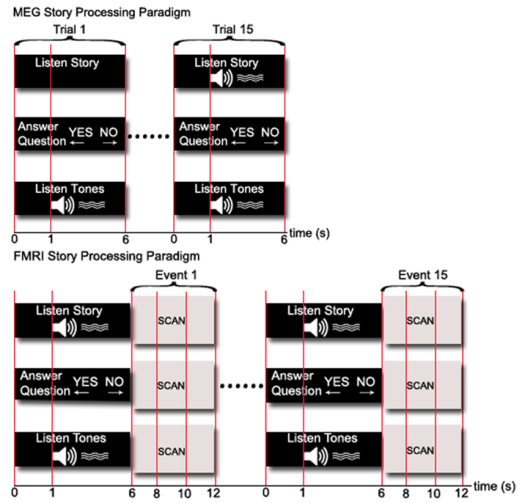


Fig. S3. No add-on noise: source reconstruction results for four different inversion schemes including no priors, all priors, valid only, and invalid only.


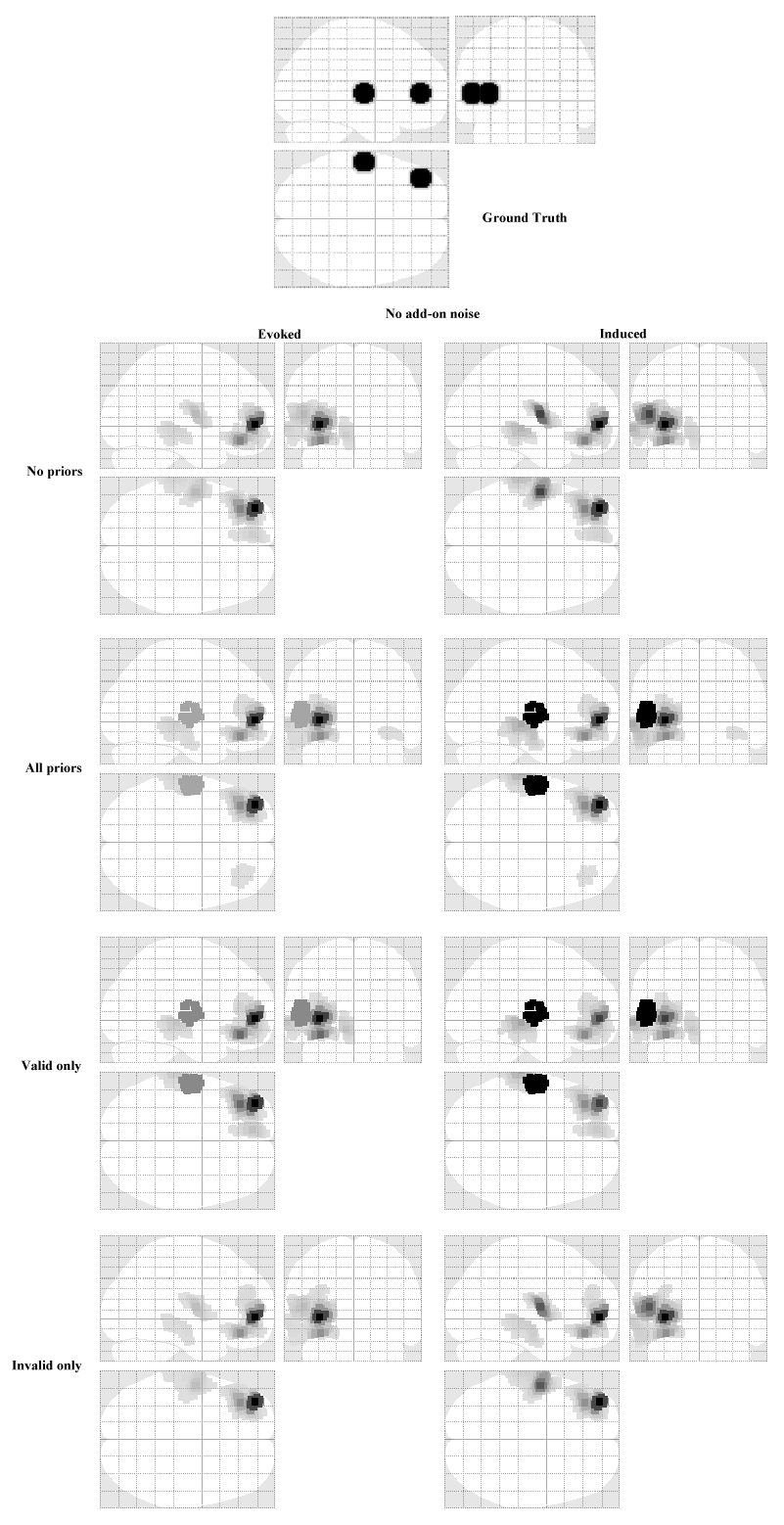


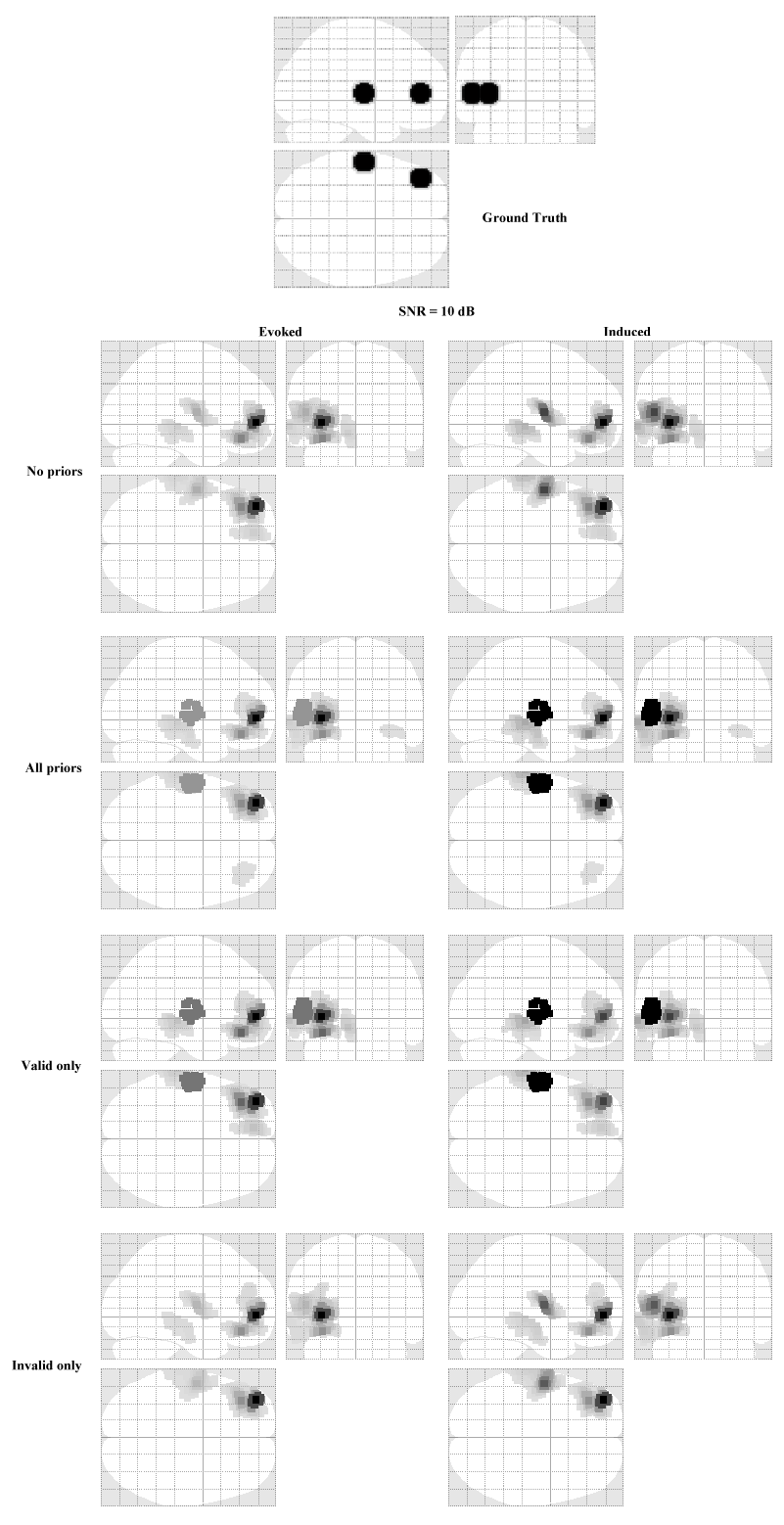
Fig. S4. SNR = 10 dB: source reconstruction results for four different inversion schemes including no priors, all priors, valid only, and invalid only.

Fig. S5. SNR = -10 dB: source reconstruction results for four different inversion schemes including no priors, all priors, valid only, and invalid only


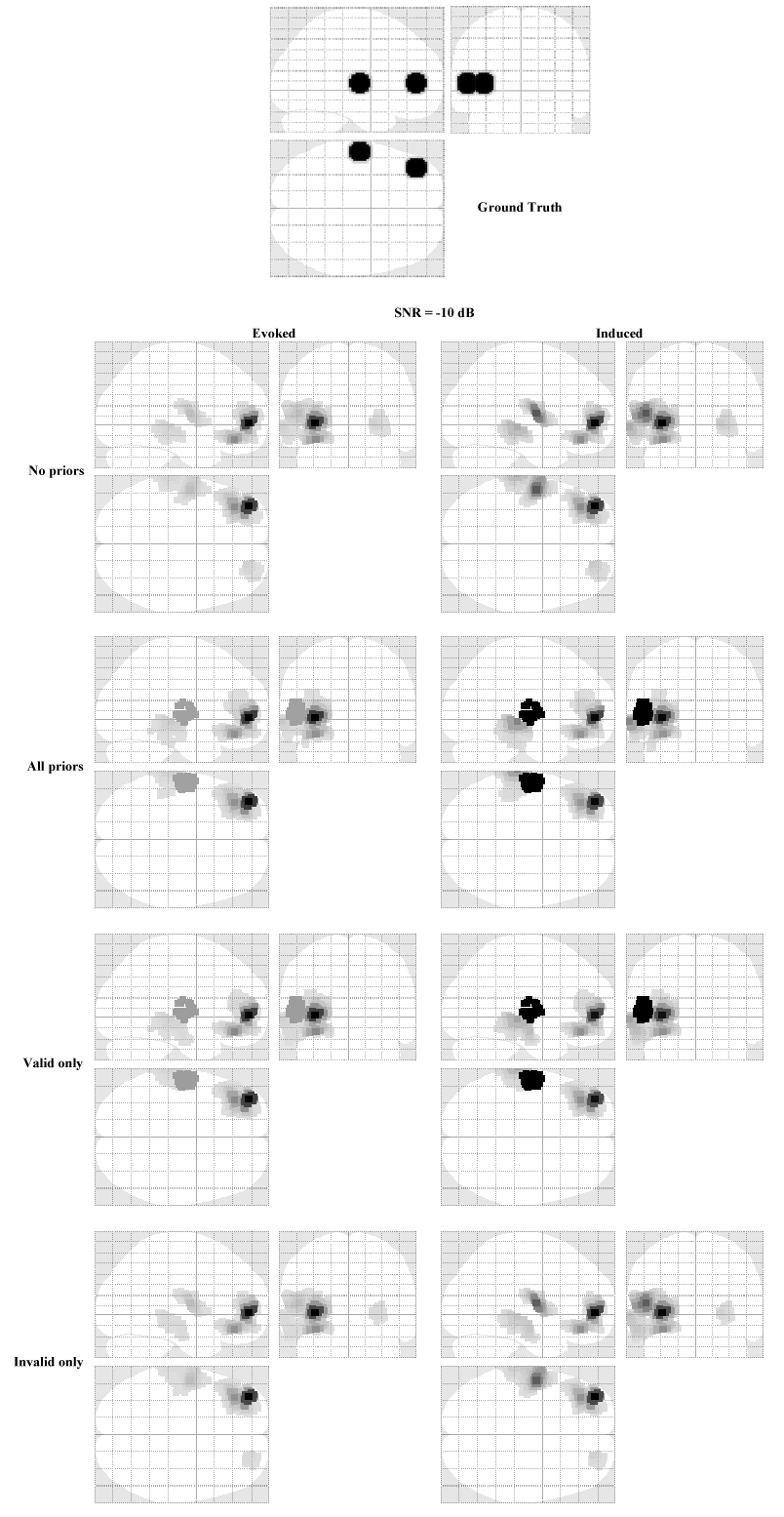


Fig. S6. Time course plots: (A1): 100 trials for source *X*_1_ (-36, 52, 2) were averaged and color coded by the type of inversion method. (A2): Single trial (#1) for source *X*_1_ (-36, 52, 2) was color coded by the type of inversion method. (B1): 100 trials for source *X*_2_ (-58, -16, 2) were averaged and color coded by the type of inversion method. (B2): Single trial (#1) for source *X*_2_ (-58, -16, 2) was color coded by the type of inversion method. Red: Ground Truth; Blue: Inversion without fMRI spatial priors; Green: Inversion with all spatial priors including valid and invalid; Cyan: Inversion only with valid fMRI spatial priors; Magenta: Inversion with invalid spatial priors; Yellow: Virtual sensor technique from Beamformer.


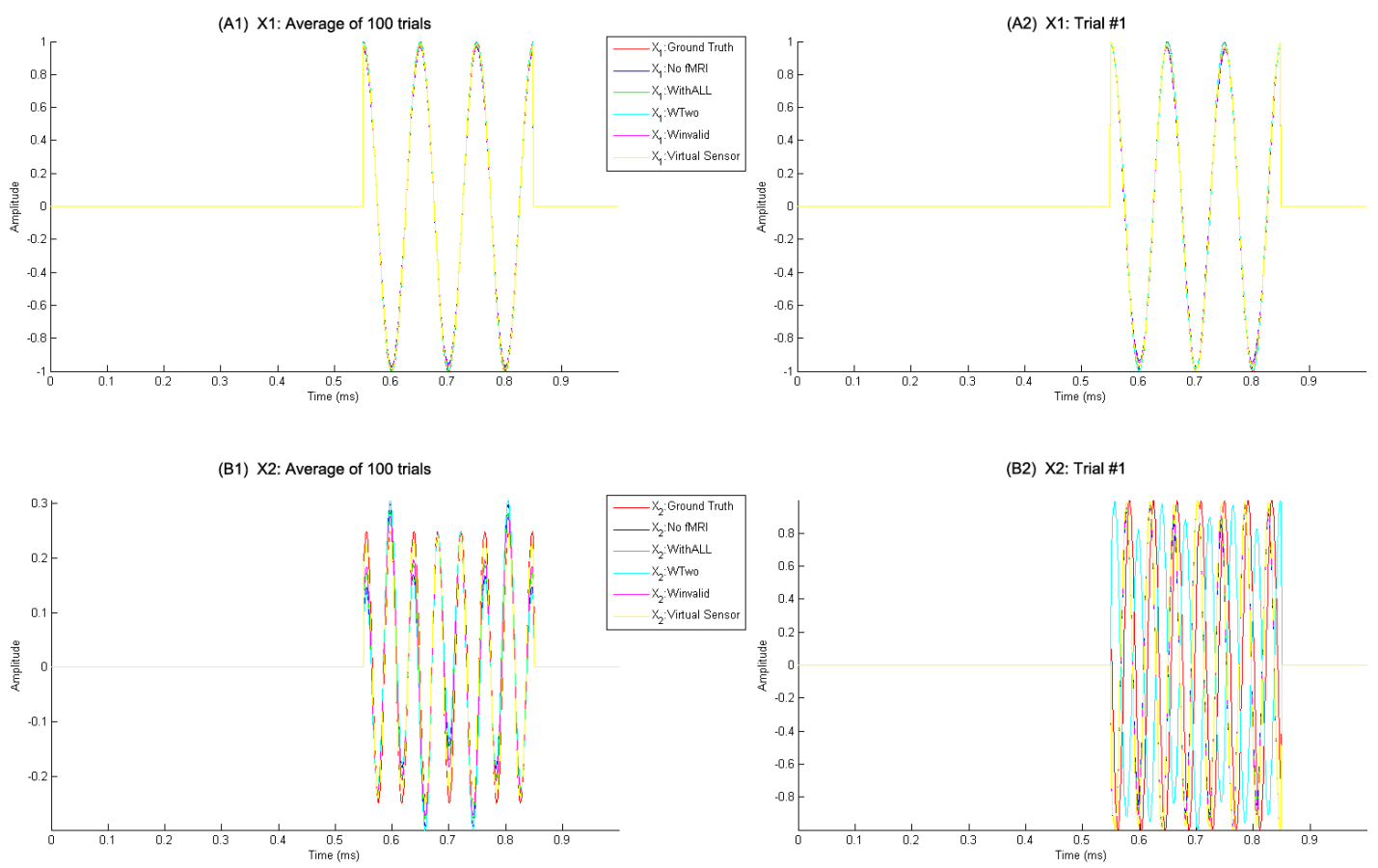


Fig. S7. Scatter plots: the actual time courses for each time point and each trial were overlapped and plotted against the extracted time courses. The x axis represents the amplitude of the actual time courses that we constructed. The y axis represents the amplitude of the extracted time courses that we estimated. Blue: Inversion without fMRI spatial priors; Green: Inversion with all spatial priors including valid and invalid; Cyan: Inversion only with valid fMRI spatial priors; Magenta: Inversion with invalid spatial priors; Yellow: Virtual sensor technique from Beamformer.


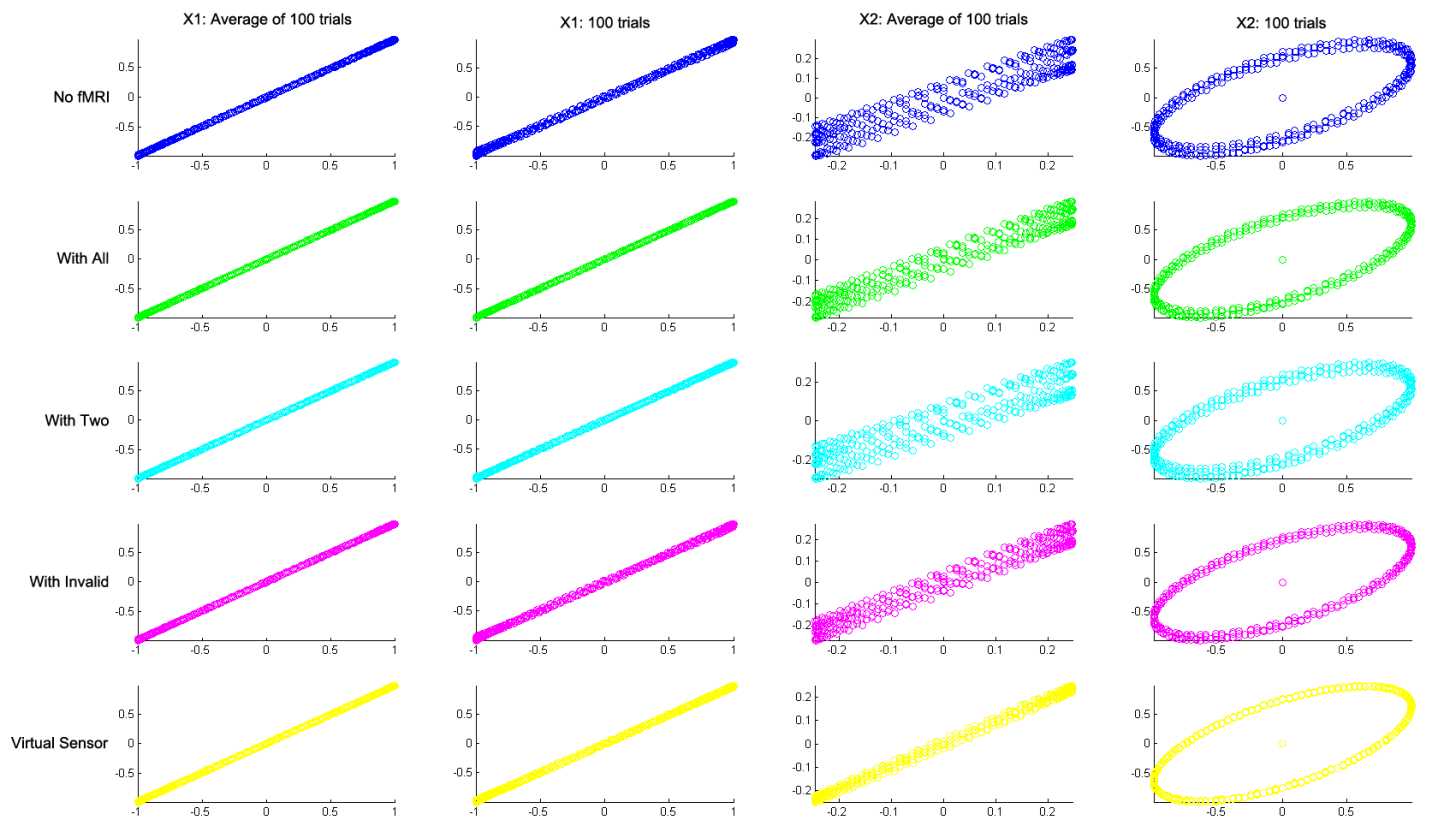


Fig. S8. Single-sided amplitude spectrum plots: x axis is the frequency range from 0 to 128 Hz and y axis is the single-sided amplitude of the power spectrum. Blue: Inversion without fMRI spatial priors; Green: Inversion with all spatial priors including valid and invalid; Cyan: Inversion only with valid fMRI spatial priors; Magenta: Inversion with invalid spatial priors; Yellow: Virtual sensor technique from Beamformer.


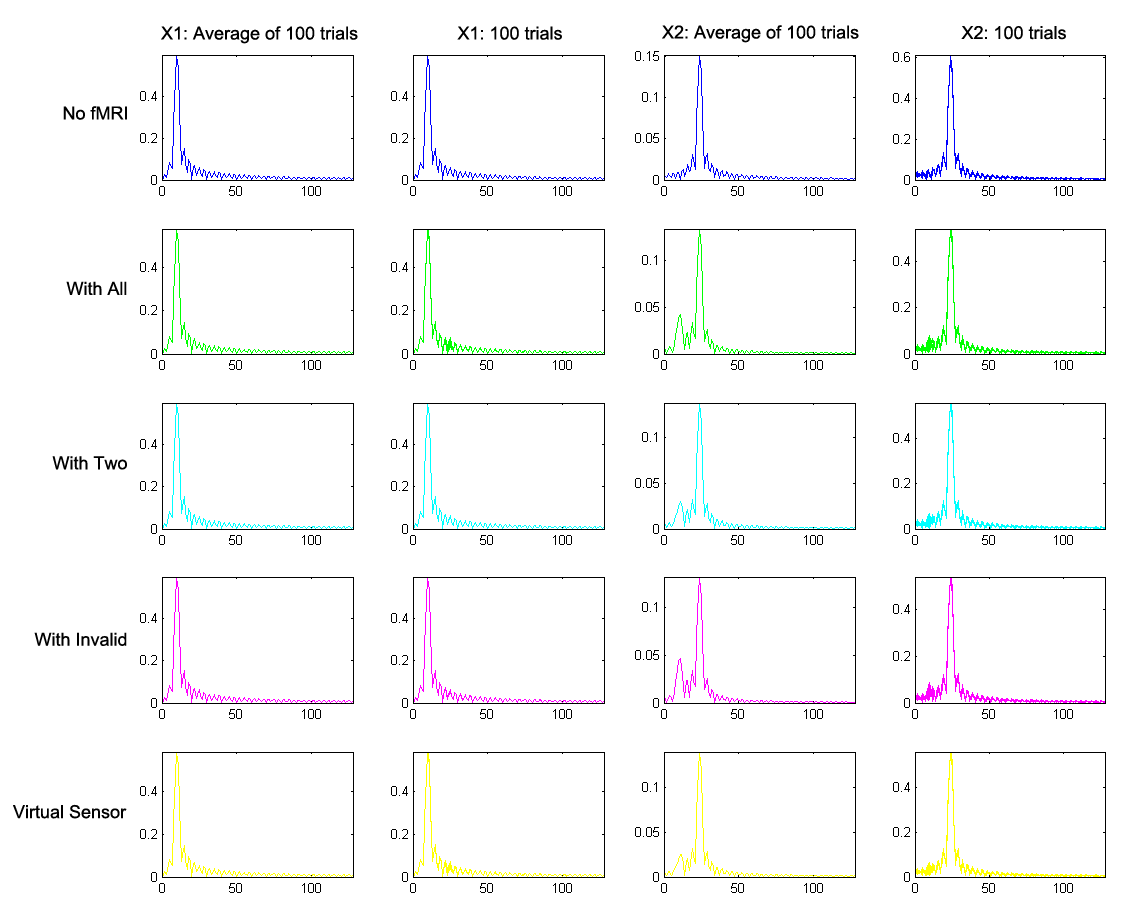


1. [↑](#footnote-ref-1)
